# **Measurement of sedentary time and physical activity in rheumatoid arthritis: an ActiGraph and activPAL**™ **validation study**

**DOI:** 10.1007/s00296-020-04608-2

**Published:** 2020-05-29

**Authors:** Ciara M. O’Brien, Joan L. Duda, George D. Kitas, Jet J. C. S. Veldhuijzen van Zanten, George S. Metsios, Sally A. M. Fenton

**Affiliations:** 1 School of Sport, Exercise and Rehabilitation Sciences, University of Birmingham, Edgbaston, Birmingham, UK; 2 Department of Rheumatology, Russells Hall Hospital, Dudley Group NHS Foundation Trust, West Midlands, UK; 3 Faculty of Education, Health and Wellbeing, University of Wolverhampton, Wolverhampton, UK

**Keywords:** Activity trackers, Physical activity, Sedentary behaviour, Rheumatoid arthritis

## Abstract

Accurate measurement of sedentary time and physical activity (PA) is essential to establish their relationships with rheumatoid arthritis (RA) outcomes. Study objectives were to: (1) validate the GT3X+ and activPAL3^μ^™, and develop RA-specific accelerometer (count-based) cut-points for measuring sedentary time, light-intensity PA and moderate-intensity PA (laboratory-validation); (2) determine the accuracy of the RA-specific (vs. non-RA) cut-points, for estimating free-living sedentary time in RA (field-validation). *Laboratory-validation*: RA patients (*n* = 22) were fitted with a GT3X+, activPAL3^μ^™ and indirect calorimeter. Whilst being video-recorded, participants undertook 11 activities, comprising sedentary, light-intensity and moderate-intensity behaviours. Criterion standards for devices were indirect calorimetry (GT3X+) and direct observation (activPAL3^μ^™). *Field-validation*: RA patients (*n* = 100) wore a GT3X+ and activPAL3^μ^™ for 7 days. The criterion standard for sedentary time cut-points (RA-specific vs. non-RA) was the activPAL3^μ^™. Results of the laboratory-validation: GT3X—receiver operating characteristic curves generated RA-specific cut-points (counts/min) for: sedentary time = ≤ 244; light-intensity PA = 245–2501; moderate-intensity PA ≥ 2502 (all sensitivity ≥ 0.87 and 1-specificity ≤ 0.11). ActivPAL3^μ^™—Bland–Altman 95% limits of agreement (lower–upper [min]) were: sedentary = (− 0.1 to 0.2); standing = (− 0.7 to 1.1); stepping = (− 1.2 to 0.6). Results of the field-validation: compared to the activPAL3^μ^™, Bland–Altman 95% limits of agreement (lower–upper) for sedentary time (min/day) estimated by the RA-specific cut-point = (− 42.6 to 318.0) vs. the non-RA cut-point = (− 19.6 to 432.0). In conclusion, the activPAL3^μ^™ accurately quantifies sedentary, standing and stepping time in RA. The RA-specific cut-points offer a validated measure of sedentary time, light-intensity PA and moderate-intensity PA in these patients, and demonstrated superior accuracy for estimating free-living sedentary time, compared to non-RA cut-points.

## Introduction

Research evidence supports the benefits of physical activity (PA) for improving health-related outcomes among people with rheumatoid arthritis (RA) [1]. More recently, studies also suggest sedentary behaviour (waking behaviour ≤ 1.5 metabolic equivalents [METs], whilst in a sitting, reclining or lying posture) [2, 3] is adversely associated with RA outcomes [4]. However, most evidence regarding the role of sedentary time and PA in RA is based on studies employing self-report methods to quantify engagement in these behaviours [4, 5].

Device-based assessments of sedentary time and PA offer a more objective measure of behaviour, and have demonstrated higher validity and reliability relative to self-report instruments [6-8]. Consequently, devices are being more readily used to measure sedentary time and PA in different populations, including in RA [4, 9]. Currently, hip-worn accelerometers (e.g., ActiGraph [Florida, USA]) are the most commonly employed device in RA studies to estimate the frequency, intensity and duration of free-living behaviour. The accelerometer records and stores raw acceleration data (g), which is subsequently processed to provide estimates of sedentary behaviour and PA. Currently, several processing methods can be applied to raw accelerometer data, with the dominant approach being the use of thresholds or ‘cut-points’ that classify behaviour as sedentary, light-intensity PA (LPA), moderate-intensity PA (MPA) or vigorous-intensity PA. There is an absence of a consensus on the ‘best’ method, with this decision dependent on the research question, study resources and research team expertise [10].

A popular and widely accessible data processing method generates sedentary time and PA estimates by applying cut-points to accelerometer activity counts (‘count-based cut-points’) that have been derived from raw accelerometer data using the device manufacturer’s proprietary software. These count-based cut-points are commonly employed, largely due to intuitive and easy-to-use software platforms that facilitate straightforward processing and analysis of complex raw accelerometer data, thus, making the application of accelerometry accessible to researchers from a wide range of disciplines (e.g., clinical/medicine, exercise science, behavioural science). However, whilst the advantages of accelerometry (and specifically, count-based cut-points) to measure sedentary time and PA are being increasingly recognised by RA researchers, several limitations exist regarding their application in this patient group.

First, few accelerometers have been specifically validated for measurement of sedentary time and PA in RA (e.g., against indirect calorimetry). Consequently, existing RA studies employing accelerometers have largely employed count-based cut-points developed in validation studies of healthy participants [11, 12] to quantify sedentary time and PA in RA. However, as RA patients differ markedly to people without RA in terms of physiology, physical function and associated activity patterns (e.g., RA patients demand a relatively higher basal metabolic rate compared to the general population [13]), such sedentary time and PA estimates should be interpreted with caution.

Second, most existing count-based cut-points are uniaxial, generating sedentary time and PA estimates using data captured by a single axis of movement. Technological advancements are such that triaxial accelerometers are now common place, and can capture data across three axes (*Y*, *X* and *Z*) to provide a more valid assessment of behaviour [14]. Thus, given the increasing popularity of applying count-based cut-points to examine sedentary time and PA in RA studies, there is a critical need to develop RA-specific triaxial accelerometer count-based cut-points to provide a valid and accessible accelerometer data processing method for RA researchers.

Still, a key limitation of accelerometers is their inability to distinguish sitting (sedentary behaviour) from standing without movement (LPA). Specifically, accelerometers work on the basis that all movements registered below a ‘sedentary time cut-point’ are by default, classed as sedentary [15]. However, low-movement behaviours may occur in a sitting or standing posture, but both may record accelerations that register below the ‘sedentary time cut-point’. Thus, accelerometers may lead to an overestimation of sedentary time by misclassifying low-movement standing behaviours as sitting (sedentary). The activPAL™ (PAL Technologies, Glasgow, UK) addresses this limitation, and is able to accurately classify behaviours as sitting/lying (sedentary), standing or stepping. This device is currently considered the gold standard for measurement of free-living sedentary time [6]. Thus, the activPAL™ primarily offers a measure of sedentary behaviour, rather than frequency, intensity and duration of PA. Consequently, few RA studies have employed the activPAL™, with extant research employing this device focusing specifically on the role of sedentary behaviour [16].

Considering exponential growth in research centred on the role of sedentary behaviour and PA for improving RA disease outcomes, it is critical that device-based measures are properly validated for use in this population. Therefore, the overarching aim of the current study was to validate the commonly employed ActiGraph GT3X+ and the activPAL3^μ^™, for measurement of sedentary time and PA in RA. In a laboratory-validation (objective 1), this study aimed to: (a) validate the GT3X+ against indirect calorimetry to generate RA-specific triaxial (vector magnitude [VM]) accelerometer count-based cut-points for sedentary time, LPA and MPA; (b) validate the activPAL3^μ^™ against direct observation for measurement of sedentary, standing and stepping time. Then, using these data, conduct a field-validation (objective 2) to compare the validity of the new RA-specific triaxial sedentary time count-based cut-point vs. a widely used non-RA uniaxial sedentary time count-based cut-point (< 100 counts/min) [11, 12] for measurement of free-living sedentary time in RA, against the gold standard (activPAL3^μ^™).

## Materials and methods

### Participants and recruitment

Participants were recruited from outpatient clinics at Russells Hall Hospital (Dudley Group NHS Foundation Trust). The only requirements for inclusion in this study were a clinical diagnosis of RA according to the American College of Rheumatology/European League Against Rheumatism classification criteria [17], and aged ≥ 18 years. For objective 1, patients were required to ambulate independently. For objective 2, patients were eligible if they could ambulate independently, or with an assistive device. Participants were excluded from objectives 1 and 2 if they were pregnant. Eligibility criteria were intentionally broad in order that the GT3X+ and activPAL3^μ^™ were validated in a more diverse population of people living with RA (e.g., males and females; low, moderate and high disease activity). All participants provided written informed consent. This study was approved by the local National Health Service Research Ethics Committee (16/WM/0371).

### Protocol

The protocol for this study has been previously published [18], but methods and analytical approaches are briefly described herein.

### Objective 1 (laboratory-validation)

Participants (*n* = 22) reported to the laboratory following a 12-h fast, and having refrained from exercise for 48 h. Upon arrival, participants completed physical assessments (e.g., height, weight, body-mass index), and underwent routine clinical evaluations to determine their disease activity (disease activity score-28 [19]) and level of functional disability (health assessment questionnaire [20]). Participants were then fitted with the GT3X+, activPAL3^μ^™, heart rate monitor (Polar Electro Oy Ltd., Kempele, Finland) and Cortex Metalyzer® 3B (indirect calorimeter [Cortex Biophysik, Leipzig, Germany]) for the duration of the laboratory-validation. For direct observation of behaviour, a video camera was set up overlooking the laboratory. All equipment was time-synchronised.

Participants undertook 11 activities (6 standardised activities and 5 activities of daily living [ADLs]). Activities required between 1.3 and 3.5 METs (ranging from sedentary behaviour to MPA) and were 6-min in duration [21]. Five-min rest periods separated the ADLs, to allow heart rate and *V*O_2_ to return to resting levels [14, 21, 22].

### Objective 2 (field-validation)

Participants (*n* = 104) attended the laboratory to complete physical assessments and routine clinical evaluations, as per objective 1. Participants were asked to wear the GT3X+ and activPAL3^μ^™ for 7 days to assess free-living sedentary time and PA [23]. The GT3X+  was worn during all waking hours, removing for water-based activities. The activPAL3^μ^™ was worn continuously for 24 h/day.

### Measures

#### Devices

The GT3X+ is a triaxial accelerometer that records accelerations on three axes (vertical [*Y*], horizontal right-left [*X*] and horizontal front-back [*Z*]), over researcher-defined time periods (epochs). These data are used to compute VM [VM = √(axis*Y*^2^ + axis*X*^2^ + axis*Z*^2^)], which is used to quantify sedentary time and PA. The GT3X+ accelerometers were set to sample movement in 1-s epochs at a rate of 30 Hz. For objectives 1 and 2, participants wore the GT3X+ attached to an elastic belt on their right hip [12, 22, 24, 25].

The activPAL3^μ^™ is an accelerometer with inclinometer function, that measures free-living behaviour over consecutive 24-h periods. For objectives 1 and 2, the activPAL3^μ^™ was worn in a mid-anterior position on the right thigh, attached with a waterproof adhesive dressing [26].

#### Criterion standards

Indirect calorimetry was the criterion standard for validating the GT3X+. The Cortex Metalyzer® 3B uses a breath-by-breath system to directly measure an individual’s concentration of inspired oxygen (O_2_) and expired carbon dioxide (CO_2_) to calculate *V*O_2_ (ml/kg/min) and METs, using MetaSoft^®^ (Cortex Biophysik). Direct observation (via video camera) was the criterion standard for validating the activPAL3^μ^™.

Following laboratory-validation of the activPAL3^μ^™, this device was employed as the criterion standard for assessing the accuracy of the RA-specific triaxial vs. the non-RA uniaxial sedentary time count-based cut-point. This decision was based on prior studies demonstrating high validity of the activPAL3^μ^™ for estimating free-living sedentary time in RA, recognising this device as the current gold standard for measurement of free-living sedentary time [6].

## Data reduction and statistical analysis

### Objective 1 (laboratory-validation)

#### GT3X+ and indirect calorimetry

The manufacturer’s software (Actilife [ActiGraph]) was used to download time-stamped GT3X+ data in the format of triaxial (VM) activity counts. Data were downloaded in counts/s, and converted to counts/min for analysis.

Metasoft^®^ was used to download and export breath-by-breath *V*O_2_ data from the Cortex Metalyzer^®^ 3B. In Microsoft Excel, second-by-second *V*O_2_ data were averaged across each minute to compute average *V*O_2_ (ml/kg/min) per minute of activity. These data were graphed to identify when steady-state *V*O_2_ was achieved within each activity (steady-state = variation within ± 0.5 ml/kg/min). Graphed data indicated steady-state occurred in min 4–6 of each activity (the final 2 min of the 11 activities). *V*O_2_ (ml/kg/min) and GT3X+ (counts/min) data were therefore averaged across min 4–6 of each laboratory testing component, to provide steady-state *V*O_2_ and GT3X+ data for each activity. These data were exported into SPSS (Chicago, USA, v.24) for statistical analysis. Where participants did not reach steady-state *V*O_2_ during an activity, their data recorded for that particular activity were excluded.

##### Statistical analysis

Average (steady-state) *V*O_2_ data were converted into METs (1 MET = 3.5 ml/kg/min) and then classified as sedentary (≤ 1.5 METs), LPA (1.6–2.9 METs) or MPA (≥ 3 METs). Using these classifications, data were recoded to create binary variables for use in receiver operating characteristic (ROC) curve analysis, to define RA-specific triaxial (VM) accelerometer count-based cut-points for sedentary time, LPA and MPA. Specifically, data were recoded as sedentary/not sedentary or MPA/not MPA using binary indicators (1/0). ROC curves identified the VM activity count maximising sensitivity (*Y-*axis) and specificity (*X-*axis) for correctly classifying behaviour as sedentary or MPA. Area under the curve (AUC) values were also calculated (AUC criteria: 0.90–1.00 = excellent; 0.80–0.89 = good; 0.70–0.79 = fair; 0.60–0.69 = poor; < 0.60 = failure).

#### ActivPAL3^μ^™ and direct observation

PAL Connect (PAL Technologies) was used to download and export activPAL3^μ^™ time-stamped data to Microsoft Excel. Outputs displayed sedentary, standing and stepping time, and number of steps and sit-stand transitions, for consecutive 15-s periods for the duration of the laboratory-validation. For direct observation, the researcher observed all video camera recordings, recording engagement in sitting/lying (sedentary), standing or stepping, as well as counting steps and sit-stand transitions, every 15 s for each activity.

##### Statistical analysis

Means (M) and standard deviations (SD) were calculated for activPAL3^μ^™-assessed and directly observed sedentary, standing and stepping time (min), and steps and sit-stand transitions (number). Bland–Altman analysis calculated 95% limits of agreement (LOA [lower to upper]) between activPAL3^μ^™-assessed vs. directly observed behaviours, using the M and SD of the differences (min) between the two measures [M ± (SD × 1.96)] [27, 28]. Finally, percentage accuracy for activPAL3^μ^™-assessment vs. direct observation of behaviours was computed [% accuracy = (activPAL3^μ^™ value/direct observation value) × 100].

### Objective 2 (field-validation)

Actilife was used to download 7-day GT3X+ data (1-s epochs) and check non-wear (criteria = ≥ 60 min of consecutive zero counts, spike tolerance of 2 min) [9, 12]. All non-wear periods identified were excluded from each participant’s data file. After removing non-wear periods, participants’ 7-day GT3X+ data were retained for inclusion in statistical analysis where GT3X+ accelerometers were worn for ≥ 10 h/day on ≥ 4 days (including ≥ 1 weekend day) [9, 12]. The RA-specific triaxial (VM) accelerometer count-based cut-point (developed in objective 1) and non-RA uniaxial (*Y*-axis) accelerometer count-based cut-point (< 100 counts/min) [11, 12], were then applied to 7-day GT3X+ data to derive estimates of free-living sedentary time (min/day).

For the activPAL3^μ^™, PAL Connect was used to download and export daily movement data (15-s epochs) that corresponded to valid days measured via the GT3X+ . Sleep time was manually removed from activPAL3^μ^™ data using wear-time logbooks and sleep-periods identified from GT3X+ data analysis. Estimates of free-living activPAL3^μ^™-assessed sedentary time (min/day) were calculated using PAL Connect proprietary algorithms.

**Statistical analysis** For objective 2, Bland–Altman analysis was used to calculate 95% LOA (lower to upper) between GT3X+- and activPAL3^μ^™-assessed free-living sedentary time, for both RA-specific and non-RA count-based cut-points. LOA were determined using the M and SD of the differences (min/day) between estimates of GT3X +- and activPAL3^μ^™-assessed sedentary time [*M* ± (SD × 1.96)].

## Results

### Objective 1 (laboratory-validation)

Twenty-two patients (86% female, *n* = 19) participated in the laboratory protocol (Table 1). *GT3X+ and indirect calorimetry:* Table 2 reports the *M* (SD) for GT3X+ activity counts and METs during steady-state *V*O_2_. Activity intensities (METs) reflecting sedentary, LPA and MPA were achieved as intended. Table 3 reports results of ROC curve analysis and the RA-specific triaxial (VM) accelerometer count-based cut-points maximising sensitivity and specificity. The AUC demonstrated ‘excellent’ fit for RA-specific sedentary time (AUC = 1.00) and MPA (AUC = 0.94) count-based cut-points. *ActivPAL3*^*μ*^*™ and direct observation:* Table 4 reports the M (SD) for activPAL3^μ^™-assessed and directly observed behaviours during the laboratory testing procedure. Compared to direct observation, the activPAL3^μ^™ accurately classified sedentary, standing and stepping time, and step number, > 98% of the time. For number of sit-stand transitions, classification accuracy was 72%.

**Table 1:** Objectives 1 and 2: participant characteristics

	Objective 1	Objective 2
Age (years)	53.7 (12.5)	58.5 (12.1)
BMI (kg/m^2^)	27.4 (5.7)	28.9 (6.1)
Height (m)	1.7 (0.1)	1.6 (0.1)
Weight (kg)	74.9 (18.0)	80.0 (20.5)
Body fat (%)	34.6 (9.3)	35.6 (8.5)
RA duration (years)	6.7 (6.3)	10.6 (10.5)
DAS-28	3.2 (1.7)	4.0 (1.5)
HAQ	0.8 (0.6)	1.2 (0.8)

M (SD) shown for age, BMI, height, weight, body fat percentage, RA duration, DAS-28 and HAQ score. DAS-28 was calculated using erythrocyte sedimentation rate, 28 swollen-and-tender joint count and visual analogue scale (overall health from 0 [very good] to 100 [very poor]). HAQ scores were defined as, ability to undertake activities of daily living (0, without any difficulty; 1, with some difficulty; 2, with much difficulty; 3, unable to do)

*BMI* body-mass index, *RA* rheumatoid arthritis, *DAS-28* disease activity score-28, *HAQ* health assessment questionnaire

**Table 2:** Objective 1: descriptive statistics for laboratory-validation of the ActiGraph GT3X+

Activity (METs)	*n*	GT3X+ (VM activity counts/min)	Energy expenditure (METs)
Standardised testing component 1			
Lying (1.3)	20	0 (0)	0.6 (0.2)
Sitting (1.3)	22	0 (0)	0.7 (0.2)
Standing (1.3)	18	141 (45)	0.8 (0.2)
Activities of daily living			
Reading a newspaper (1.3)	19	7 (13)	0.8 (0.2)
Washing and drying dishes (1.8)	15	518 (315)	1.8 (0.3)
Ironing and folding clothes (2.0)	12	549 (279)	1.9 (0.3)
Placing bed linens on pillows and duvet (2.5)	18	1051 (526)	2.3 (0.5)
Sweeping the floor (3.3)	17	1675 (502)	2.3 (0.6)
Standardised testing component 2			
Walking at 3.2 km/h (2.8)	19	2148 (571)	2.7 (0.7)
Walking at 4 km/h (3.0)	20	3120 (637)	3.2 (0.8)
Walking at 4.8 km/h (3.5)	18	3944 (882)	3.4 (0.4)

MET values (compendium of physical activities [35]) are specified next to each activity. M (SD) are shown for GT3X+ activity counts (VM) and METs, averaged across min 4–6 of each activity. Number of participants (*n*) included in analysis are shown per activity

*METs* metabolic equivalents, *VM* vector magnitude

**Table 3:** Objective 1: ROC curve-generated RA-specific triaxial (VM) accelerometer count-based cut-points

Epoch (1-min)	RA-specific count-based cut-points (VM activity counts/min)	Sensitivity	1-Specificity	AUC
Sedentary time	≤ 244	0.99	0.03	1.00
LPA	> 244–< 2502	–	–	–
MPA	≥ 2502	0.87	0.11	0.94

RA-specific count-based cut-points were developed for sedentary time, LPA and MPA, based on average GT3X+ activity counts (VM) and METs during steady-state *V*O_2_ (± 0.5 ml/min/kg [min 4–6 of each activity]). LPA count-based cut-points were defined using the upper cut-point threshold of sedentary time and the lower cut-point threshold of MPA. AUC demonstrated accuracy of the RA-specific count-based cut-points (0.90–1.00 = excellent; 0.80–0.89 = good; 0.70–0.79 = fair; 0.60–0.69 = poor; < 0.60 = failure)

*RA* rheumatoid arthritis, *VM* vector magnitude, *AUC* area under the curve, *LPA* light-intensity physical activity, *MPA* moderate-intensity physical activity, – does not apply

**Table 4:** Objective 1*:* descriptive statistics for laboratory-validation of the activPAL3^μ^™

	ActivPAL3^μ^™	Direct observation	Accuracy (%)
Sedentary (total min)	18.1 (0.1)	18.0 (0.0)	99.6
Standing (total min)	29.2 (0.8)	28.9 (0.6)	99.2
Stepping (total min)	18.8 (0.8)	19.1 (0.6)	98.4
Steps (total number)	2044 (122)	2074 (144)	98.6
Sit-stand transitions (total number)	5 (1)	7 (0)	72.1

M (SD) are shown for total activPAL3^μ^™-assessed and directly observed time spent sedentary, standing and stepping (total min), and number of steps and sit-stand transitions, during each activity of the laboratory protocol. The percentage accuracy for activPAL3^μ^™-assessment vs. direct observation of each behaviour is also shown [% accuracy = (activPAL3^μ^™ value/direct observation value) × 100]

**Fig. 1:**
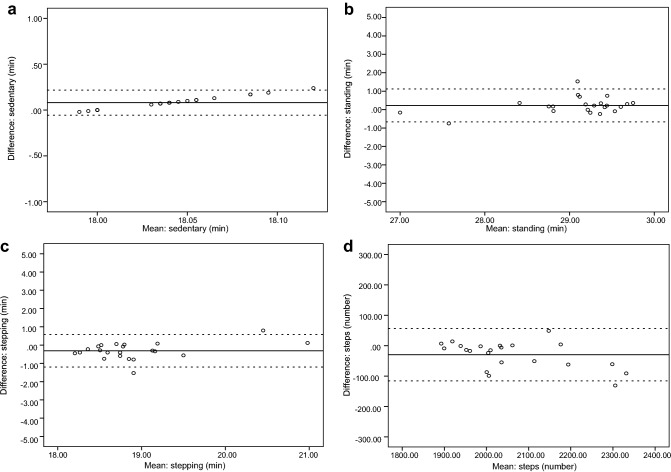
Objective 1: Bland–Altman plots showing agreement (mean difference and 95% limits of agreement [LOA]) for time spent sedentary (**a**), standing (**b**), and stepping (**c**), as well as number of steps (**d**), between the activPAL3^μ^™ vs. direct observation. Note: Straight full line represents mean difference and the straight dotted line represents lower and upper LOA (95%)

Mean differences for activPAL3^μ^™-assessed vs. directly observed behaviours were computed (M [SD]): sedentary time = 0.1 (0.1) min; standing = 0.2 (0.5) min; stepping = − 0.3 (0.5) min; steps = − 30 (44); sit-stand transitions = − 2 (1). Bland–Altman analysis (Fig. 1) demonstrated narrow 95% LOA (lower to upper) for sedentary (− 0.1 to 0.2), standing (− 0.7 to 1.1) and stepping (− 1.2 to 0.6) time (min). For number of steps, 95% LOA were wider (− 116 to 57). As only M (SD) = 5 (1) and M (SD) = 7 (0) sit-stand transitions were recorded by the activPAL3^μ^™ and direct observation, respectively, Bland–Altman plots could not be produced for this outcome.

### Objective 2 (field-validation)

A total of *n* = 100 participants (96% [71% female, *n* = 71]) provided valid 7-day GT3X+ and corresponding activPAL3^μ^™ data (Table 1). GT3X+-derived sedentary time estimates (M [SD]) were: RA-specific count-based cut-point = 686.1 (72.4) min/day vs. non-RA count-based cut-point = 754.7 (62.5) min/day.

**Fig. 2:**
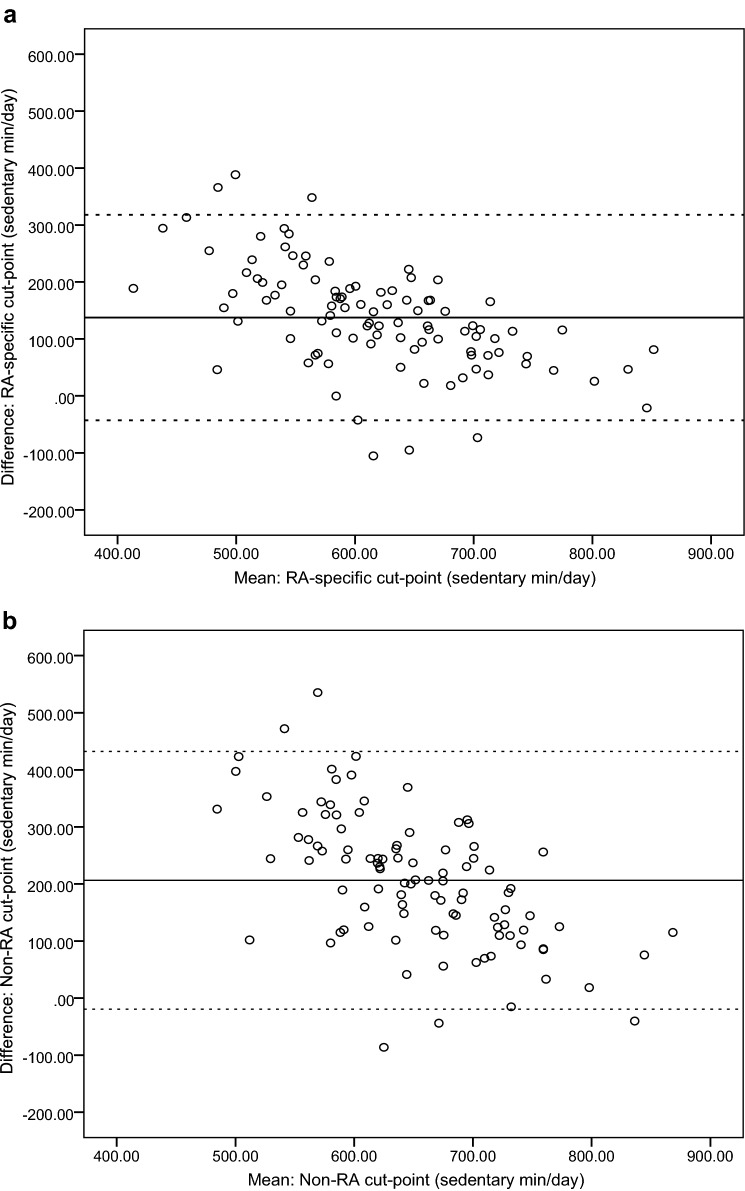
Objective 2: Bland–Altman plots showing agreement (mean difference and limits of agreement [LOA]) between GT3X+-assessed vs. activPAL3^μ^™-assessed sedentary time. Accelerometer count-based cut-points applied were: RA-specific (VM) count-based cut-points [≤ 244 count/min, derived from objective 1 of this study (**a**)], and non-RA (*Y*-axis) count-based cut-points [< 100 counts/min (**b**)]. Note: Straight full line represents mean difference and the straight dotted line represents lower and upper LOA (95%)

For the RA-specific count-based cut-point (≤ 244 counts/min) vs. the activPAL3^μ^™, Bland–Altman analysis (Fig. 2) revealed a mean difference of 137.7 (SD = 92.0), with 95% LOA (lower to upper) = (− 42.6 to 318.0), for sedentary time (min/day). Most data points were positioned above zero and followed a downward trend, whereby a lower mean difference between measures was observed at higher levels of sedentary time.

Compared to the RA-specific triaxial count-based cut-point, the non-RA uniaxial count-based cut-point demonstrated a greater mean difference (206.2 [SD = 115.2]) and wider 95% LOA (lower to upper) = (− 19.6 to 432.0) vs. the activPAL3^μ^™ for sedentary time (min/day). Bland–Altman analysis for the non-RA count-based cut-point revealed most data points were scattered above zero, and a downward trend was observed (lower mean difference between measures at higher levels of sedentary time).

## Discussion

The current study validated the ActiGraph GT3X+ and activPAL3^μ^™—two devices commonly used in sedentary behaviour and PA research—for measurement of sedentary time and PA in people living with RA. Whilst there are several options for processing raw accelerometer data to quantify sedentary time and PA in healthy populations, count-based cut-points offer an accessible means of accelerometer data processing for researchers and health professionals working in rheumatology. To date, RA studies employing accelerometers have largely relied on the application of non-RA count-based cut-points to quantify free-living sedentary time and PA in this population [29, 30], which are limited in their validity when we consider the unique physiology and associated movement patterns of people living with RA [21, 22, 24]. Thus, there exists a critical need for the development of RA-specific count-based cut-points, which can be easily and consistently employed across RA studies.

In response, this is the first study to calibrate the commonly employed GT3X+ and define RA-specific triaxial accelerometer count-based cut-points, for valid measurement of sedentary time, LPA and MPA in RA. Our RA-specific count-based cut-points were derived according to energy requirements of behaviour among people with RA, and demonstrated high sensitivity and specificity for classification of sedentary time, LPA and MPA. Thus, the application of our novel RA-specific triaxial count-based cut-points are likely to provide more valid assessments of sedentary time and PA in RA, relative to employing non-RA uniaxial count-based cut-points developed in validation studies of healthy adults. We therefore recommend using the RA-specific count-based cut-points proposed herein, in future RA research.

This study also assessed the accuracy of the activPAL3^μ^™ for measurement of sedentary, standing and stepping time in RA. Only one study has examined the ability of the activPAL™ to validly assess posture in RA [31]. Larkin et al. [31] employed regression analysis and observed strong *associations* between activPAL™-assessed sedentary, standing and stepping time with directly observed behaviour. However, it would be surprising to find a non-significant relationship between two methods designed to measure the same variables [27, 28]. Thus, we employed Bland–Altman analysis to determine *agreement* between activPAL3^μ^™-assessed vs. directly observed behaviours [27, 32], and reported high classification accuracy (> 98%) between the two measures for all behaviours, in our sample of RA patients. This is in line with past research in non-RA populations [26, 33] and further supports the recommendation that the activPAL™ be considered the gold standard for assessment of free-living sedentary time [6], including in RA.

On the basis of this recommendation, we examined the validity of the RA-specific sedentary time count-based cut-point, using the activPAL3^μ^™ as the criterion standard. Results revealed a mean difference of 2.3 h/day between sedentary time quantified using the RA-specific count-based cut-point vs. the activPAL3^μ^™. Bland–Altman plots demonstrated most data points to fall above zero, suggesting overestimation of sedentary time using the RA-specific count-based cut-point, compared to the activPAL3^μ^™. Still, when compared to the activPAL3^μ^™, our RA-specific count-based cut-point produced a smaller mean difference, and narrower 95% LOA, relative to the commonly used non-RA count-based cut-point (< 100 counts/min) [11, 12].

It is possible that the observed lack of agreement between sedentary time quantified using the RA-specific count-based cut-point vs. activPAL3^μ^™-assessed sedentary time in this study reflects the inability of accelerometers to differentiate between sitting and standing, rather than relatively compromised validity of the RA-specific count-based cut-point described herein. Our data support this as a plausible explanation for two reasons. First, participants’ average MET value during ‘standing’ in the laboratory protocol was 0.8 METs (< the 1.5 METs used to define sedentary behaviour). Second, the downward trend observed in Bland–Altman plots suggests agreement between GT3X+- and activPAL3^μ^™-assessed sedentary time improves at higher levels of sedentary time, where lower levels of PA (including standing) are likely to occur. That is, for people engaging in high levels of sedentary time, standing may occupy less of daily waking behaviour and, therefore, there is less opportunity to misclassify standing time as sedentary time. In a recent study comparing accelerometer- and activPAL™-assessed sedentary time in older adults, Aguilar-Farías et al. [24] demonstrated that their population-specific sedentary time VM count-based cut-points (e.g., < 60 counts/min) were better able to detect combined activPAL™-assessed sedentary and standing time (AUC = 0.82), compared to activPAL™-assessed sedentary time alone (AUC = 0.73).

In summary, results suggest that future studies should employ the activPAL3^μ^™ for valid assessment of sedentary time in people living with RA. When this is not possible, the RA-specific sedentary time count-based cut-point represents a more valid alternative, relative to the non-RA count-based cut-point of < 100 counts/min [11, 12] in this population. However, these recommendations should be considered in the context of study limitations. First, the nature of the laboratory-validation meant that a free-living environment could not be wholly achieved, only replicated. Still, the laboratory protocol was informed by similar validation studies conducted in RA and non-RA populations, and included several activities typically undertaken in a free-living environment [21, 31, 34]. Second, participants not reaching steady-state *V*O_2_ during laboratory-validation activities were excluded from ROC curve analysis, which reduced the number of data points available for cut-point calibration (out of a possible 199: sedentary time = 82; LPA = 87; MPA = 30). Nevertheless, the number of data points for each activity intensity are comparable to other studies that have developed accelerometer count-based cut-points for measuring sedentary time, LPA and MPA in populations with reduced physical function [14]. Third, participants included in both laboratory- and field-based protocols were mostly females with moderate RA disease activity. Thus, findings may be less generalisable to male RA patients and those with more/less active disease. Future research should, therefore, confirm the validity of the RA-specific count-based cut-points and activPAL3^μ^™ in different populations of RA patients (e.g., males, higher/lower disease activity). The current study has provided a ‘first step’ towards further work in this area.

Finally, the primary aim of the current study was to develop RA-specific triaxial accelerometer count-based cut-points to allow researchers to easily and consistently apply these criteria to accelerometer data in the RA population with heightened accuracy, compared to non-RA (and uniaxial) count-based cut-points. Indeed, the development of RA-specific count-based cut-points fills an important gap in the literature, providing an accessible tool for the growing number of rheumatology professionals (e.g., consultants, nurses, physiotherapists) conducting research to understand the role of sedentary time and PA in RA. However, due to a rapidly evolving field and technological advancements in the measurement of sedentary time and PA, it is important that future research examines the validity of other emerging analytical approaches that involve the development of complex data processing algorithms, to compliment the count-based cut-point validation model employed herein.

## Conclusion

This study confirms the activPAL3^μ^™ can be considered the gold standard for measurement of free-living sedentary time in RA. Further, RA-specific triaxial accelerometer count-based cut-points presented herein are sensitive and specific for measurement of sedentary time, LPA and MPA, and permit more accurate assessment of free-living sedentary time compared to the commonly employed non-RA uniaxial accelerometer count-based cut-point [11, 12]. Thus, in the absence of the activPAL3^μ^™, our data support use of the RA-specific count-based cut-point for assessment of sedentary time in this patient group.
